# Automatic Exposure Control Attains Radiation Dose Modulation Matched with the Head Size in Pediatric Brain CT

**DOI:** 10.3390/tomography8060246

**Published:** 2022-12-13

**Authors:** Yusuke Inoue, Hiroyasu Itoh, Hiroki Miyatake, Hirofumi Hata, Ryosuke Sasa, Nao Shiibashi, Kohei Mitsui

**Affiliations:** 1 Department of Diagnostic Radiology, Kitasato University School of Medicine, Sagamihara 252-0374, Kanagawa, Japan; 2 Department of Radiology, Kitasato University Hospital, Sagamihara 252-0375, Kanagawa, Japan

**Keywords:** computed tomography, radiation dose, pediatrics, brain, automatic exposure control

## Abstract

We investigated the relationship between the head size and radiation dose in pediatric brain computed tomography (CT) to evaluate the validity of automatic exposure control (AEC). Phantom experiments were performed to assess image noise with and without AEC, and indicated that AEC decreased differences in noise between slices of different section sizes. Retrospective analysis was conducted on 980 pediatric brain CT scans where the tube current was determined using AEC. The water equivalent diameter (WED) was employed as an index of the head size, and mean WED for each image set (WEDmean) and WED for each slice (WEDslice) were used for analysis. For the image-set-based analysis, volume CT dose index (CTDIvol) was compared to WEDmean. For the slice-based analysis, the tube current was compared to WEDslice using 20 of the 980 sets. Additionally, CTDIvol and WEDmean were compared between male and female patients matched for age, weight, or WEDmean. CTDIvol increased with increasing WEDmean, and an exponential curve was closely fitted to the relationship. Tube current changed similarly to the change in WEDslice for each image set, and an exponential curve was well-fitted to the plots of tube current against WEDslice when data from the 20 sets were pooled together. Although CTDIvol and WEDmean were slightly but significantly larger for male than female patients after matching for age or weight, a sex-dependent difference in CTDIvol was not found after matching for WEDmean. This study indicated successful dose modulation using AEC according to the head size for each patient and each slice location. The application of AEC to pediatric brain CT is recommended for radiation dose optimization.

## 1. Introduction

Computed tomography significantly contributes to patient care in contemporary medicine; however, its high radiation dose is a significant concern. In pediatric clinical practice, the main application of CT is the evaluation of head abnormalities [1,2]. A high morbidity of brain tumors has been reported for children who underwent brain CT [3,4,5]. Children are more radiosensitive than adults, and their long expected lifetime allows the development of cancer after a long latency period. Therefore, justification and optimization in pediatric brain CT are essential [6].

For optimization in radiological imaging, radiation dose is reduced while keeping image quality acceptable for diagnostic purposes. In CT, the patient is exposed to X-ray photons. Photons are attenuated by the patient, and only a small fraction of the photons reach the detector and are utilized for image reconstruction. When an image section is larger and X-ray attenuation is stronger, more radiation exposure is needed to avoid an excessive increase in image noise and maintain appropriate image quality. Radiation exposure in CT is proportional to the tube current and is mainly adjusted through the modulation of tube current. Automatic exposure control (AEC) modulates tube current automatically based on the degree of X-ray attenuation primarily estimated on the localizer images [7,8,9]. AEC increases the tube current in a large patient and at a highly attenuating location within a patient. This technique allows for the adjustment of the radiation dose for each patient and each location to keep image quality constant.

Radiation dose should correspond to the patient size from the aspect of optimization. In radiation dose management of CT, the volume CT dose index (CTDIvol) and dose length product, an integral of CTDIvol over the scan range, are frequently used as indices of radiation dose. The effective diameter is a basic index of the image section size and is defined as a geometric mean of the anteroposterior and lateral diameters [10]. Even when the effective diameter is identical, attenuation is stronger for a section with more bones and weaker for a section with more air. The water equivalent diameter (WED) is a more sophisticated index of the section size and is calculated considering differences in attenuation strengths among tissues [11]. Appropriate radiation dose modulation should alter tube current and CTDIvol according to the WED.

Although AEC is recognized as a valuable tool for CT dose optimization, its application to pediatric brain CT is less common than pediatric body CT, and large surveys demonstrated that about half of the facilities did not use AEC for pediatric brain CT [12,13]. Differences in head size are generally smaller than those in body size but are still present, especially among young children. AEC may aid radiation dose optimization in pediatric brain CT by adjusting the tube current according to the head size. In this study, we investigated the relationship between the WED and CTDIvol or tube current in pediatric brain CT to evaluate the validity of AEC-based dose modulation.

## 2. Materials and Methods

### 2.1. Imaging Procedures

All examinations were performed on two 64 detector-row CT scanners with the same specifications (Optima CT 660 Discovery Edition; GE Healthcare, Milwaukee, WI, USA). The head was positioned on the head holder, and posteroanterior and then lateral localizer images were acquired with a tube voltage of 120 kV, a tube current of 10 mA, a table speed of 100 mm/s, and a beam width of 5 mm. Image sections for CT were planned on the localizer images. Axial images were acquired from the caudal margin of the posterior fossa to the top of the brain in the non-helical mode, with a tube voltage of 120 kV, a rotation time of 1 s, a beam width of 10 mm, a slice thickness of 5 mm, and a slice increment of 5 mm. Tube current was determined using AEC software consisting of Auto mA and Smart mA (GE Healthcare) [8]. The noise index was set at 4 in patient imaging, and at 2.5, 3, 3.5, 4, 4.5, and 5 in phantom experiments. This index represents the noise level of CT images reconstructed using standard filtered backprojection. A higher noise index leads to weaker radiation exposure and higher noise level. Organ dose modulation (ODM) was applied in patient imaging, but not in phantom experiments, over the orbit to decrease radiation exposure from the anterior direction and consequently decrease the radiation dose to the eye lens [14,15]. CT images were reconstructed with adaptive statistical iterative reconstruction (ASiR, 60% blending) and a field of view of 250 mm.

### 2.2. Phantom Experiments

Phantom experiments were conducted to evaluate the effect of AEC-based dose modulation on image noise. The head portion of an anthropomorphic whole-body phantom (PBU-60; Kyoto Kagaku, Kyoto, Japan) was placed on the head holder and was imaged using AEC with various noise indices. Moreover, the phantom was imaged not using AEC, with a fixed tube current of 180 mA. The imaging experiments were performed in triplicate.

Two contiguous slices imaged simultaneously in one rotation were selected at the cranial and middle locations. The middle location corresponded to the level of the basal ganglia. Three circular regions of interest (ROIs) of 15 mm in diameter were selected for each cerebral hemisphere, and the standard deviations of the pixel values in Hounsfield units (HU) were obtained as image noise. The positions of the ROIs were identical among the image sets acquired with and without AEC. Image noise for each cranial and middle location was calculated from 36 ROIs (6 ROIs × 2 slices × 3 image sets). The cranial-to-middle ratio was defined as the ratio of the image noise at the cranial location to that at the middle location. Additionally, the WED was calculated for each slice using a radiation dose management system Radimetrics (Bayer Medical Care Inc., Indianola, PA, USA) and averaged between the two contiguous slices. For the WED calculation, the area of the imaging object was automatically demarcated on the CT image, and the pixel values in the area were summed together to determine the diameter of a round water disc equivalent to the imaging object in terms of X-ray attenuation. Although truncation may cause errors in body CT, it did not matter in this study regarding brain CT.

### 2.3. Patients

A total of 980 CT examinations performed in patients younger than 15 years were analyzed retrospectively. Using the age of 15 years to distinguish between children and adults is commonly used for clinical medicine in our country. Among these examinations, 544 and 436 examinations were performed in males and females, respectively, and 101, 110, 313, 239, and 217 examinations were performed at 0–<0.25, 0.25–<1, 1–<5, 5–<10, and 10–<15 years, respectively. These data were used in previous studies for different purposes [16,17]. For patients who underwent CT examinations repeatedly, those performed at an interval longer than 1 year were included in analysis. Data were excluded because of lack of body weight records (n = 52), weight over 80 kg (n = 5), use of the adult CT protocol (n = 9), helical-mode imaging (n = 8), and no use of the head holder (n = 5). Kitasato University Medical Ethics Organization (Sagamihara, Japan) approved this study (B20-114), and the need for informed consent was waived.

### 2.4. Image-Set-Based Analysis in Patients

The relationship between CTDIvol and WED was assessed on an image-set basis. CTDIvol calculated by the CT scanner was recorded for each image set. Mean WED over the scan range (WEDmean) was determined using Radimetrics. CTDIvol was plotted against WEDmean (n = 980), and exponential curve fitting was conducted.

### 2.5. Slice-Based Analysis in Patients

The relationship between tube current and WED was assessed on a slice-by-slice basis. All image sets were arranged in ascending order according to age, and 20 sets were extracted for analysis with a constant interval. The WED for each slice (WEDslice) was determined using Radimetrics, while the tube current value for each slice was retrieved from the digital imaging and communications in medicine (DICOM) header. For each image set, the tube current was plotted against WEDslice, and an exponential curve was fitted to the plots. Additionally, slice-based data of all 20 image sets were pooled together, and the relationship between tube current and WEDslice was evaluated with exponential curve fitting. Pooled analysis was also conducted excluding the most cranial slice of each image set.

### 2.6. Sex-Dependent Differences

The effect of sex on CTDIvol was assessed. Male and female groups matched for age, weight, or WEDmean were generated using propensity score-matched analysis [18,19]. The propensity score was calculated using logistic regression analysis. One-to-one nearest-neighbor matching without replacement was conducted using a caliper width of 0.2. CTDIvol and WEDmean were compared between male and female groups using the Wilcoxon rank sum test.

### 2.7. Statistical Analysis

Exponential curve fitting was conducted using Microsoft Excel (Microsoft Corp., Redmond, WA, USA). JMP Pro (Ver.16.1.0; SAS Institute Japan, Tokyo, Japan) was used for the Wilcoxon rank sum test and propensity score-matched analysis. A *p*-value of less than 0.05 was deemed statistically significant.

## 3. Results

### 3.1. Phantom Experiments

The WED was 14.8 cm and 19.0 cm for the cranial and middle locations, respectively. When the tube current was fixed at 180 mA without AEC, image noise was smaller at the cranial location than at the middle location, with the cranial-to-middle ratio of 0.75 (Table 1). When using AEC, the tube current was lower at the cranial location than at the middle location. The use of AEC reduced the differences in image noise irrespective of the noise index, with the cranial-to-middle ratio ranging from 0.92 to 0.94.

### 3.2. Image-Set-Based Analysis in Patients

**Figure 1:**
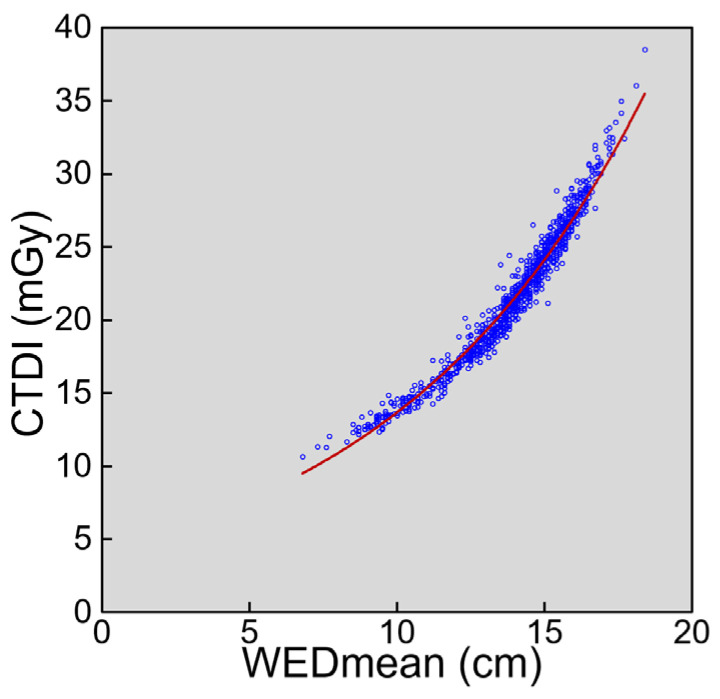
The relationship between CTDIvol and WEDmean. The red line represents an exponential regression curve (y = 4.396e^0.1135x^, R^2^ = 0.968).

The WEDmean and CTDIvol ranged widely from 6.8–18.4 cm 10.7–38.5 mGy, respectively. CTDIvol increases with increasing WEDmean (Figure 1). CTDIvol increase was accelerated with increasing WEDmean, and an exponential curve was closely fitted to the plots of CTDIvol against WEDmean.

### 3.3. Slice-Based Analysis in Patients

**Figure 2:**
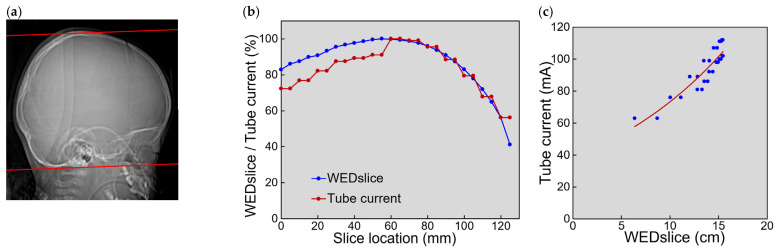
Tube current modulation in a 1.8-year-old male patient. (**a**) The localizer image is presented with red lines indicating the scan range. (**b**) WEDslice (blue) and tube current (red) are expressed as percentages of their maximum values and presented along the slice location. The slice location 0 corresponds to the most caudal slice. (**c**) The relationship between tube current and WEDslice. The red line represents an exponential regression curve (y = 38.17e^0.0653x^, R^2^ = 0.845).

The profiles of WEDslice and tube current along the slice location and the relationship between the tube current and WEDslice are exemplified in Figure 2. WEDslice increased from the caudal end to the middle level and decreased from the middle level to the cranial end. The tube current changed similarly to WEDslice, whereas tube current relative to its maximum value was lower in the caudal region than WEDslice relative to its maximum value. Tube current correlated with WEDslice. Within each of the 20 image sets, the tube current increased with increasing WEDslice. The median coefficient of determination R^2^ was 0.885 (range, 0.692–0.945).

**Figure 3:**
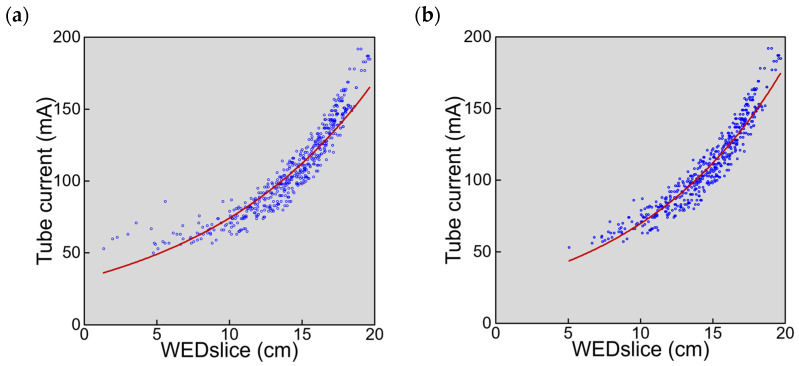
The relationships between tube current and WEDslice in 20 examinations. (**a**) Plots of data from all slices (y = 32.45e^0.0828x^, R^2^ = 0.878). (**b**) Plots excluding the most cranial slice of each image set (y = 26.98e^0.0949x^, R^2^ = 0.917). The red lines represent exponential regression curves.

Pooled analysis of the 20 image sets demonstrated that tube current increased with increasing WEDslice (Figure 3a). An exponential curve was well-fitted to the plots of tube current against WEDslice; however, plots with small WEDslice values tended to be present above the fitting curve. Excluding the most cranial slice of each image set improved exponential curve fitting (Figure 3b).

### 3.4. Sex-Dependent Differences

When matching was performed for age or weight, CTDIvol and WEDmean were slightly but significantly larger in male vs. female patients (Table 2). Matching for WEDmean eliminated sex-dependent differences in CTDIvol.

## 4. Discussion

Although the utility of AEC is widely recognized, it is not necessarily used in pediatric brain CT. An Italian survey of pediatric CT doses published in 2015 demonstrated that AEC was used in 80.5% of abdominal studies, 68.5% of chest studies, and only 50.5% of head studies despite the availability of AEC software on all CT scanners [12]. A French survey published in 2020 revealed that AEC was used in 70% to 100% of body studies and 50 to 63% of brain studies [13]. It should be noted that AEC may modulate the radiation dose unreasonably. For example, the tube current along the *z*-axis determined using a given AEC system may vary depending on the direction of the localizer imaging [20,21,22,23]. Using a widely used AEC system, a much higher tube current was observed in the shoulder region using posteroanterior localizer images than using posteroanterior and lateral images [21,22]. A markedly high dose was observed at the top of the head depending on the patient positioning, which was resolved after modification of the AEC system [24]. Appropriate dose modulation is not always guaranteed, and the validity of AEC should be investigated in clinical situations.

In this study, we evaluated the relationship between the head size and radiation dose in pediatric brain CT performed using AEC for radiation dose modulation. The AEC software used in this study estimates the strength of X-ray attenuation based on a single localizer image obtained just before CT planning (the lateral image in this study) and adjusts the tube current to keep image noise constant. When the head size is large and thus attenuation is strong, the proportion of X-ray photons that pass through the head and reach the detector is small, causing the need for high radiation exposure to maintain image quality. The image-set-based analysis demonstrated that CTDIvol increased with increasing WEDmean, indicating that AEC increased radiation exposure for patients with large heads. An exponential curve was closely fitted to the relationship, consistent with exponential X-ray attenuation through the path. The head size is relatively constant in adults but differs largely in children. Considering the wide ranges in WEDmean and CTDIvol, radiation dose modulation using AEC appears to offer substantial benefits to children who undergo brain CT.

Radiation output is proportional to tube current at a given tube voltage value. In slice-based analysis, the tube current changed similarly to WEDslice along the slice location, and an exponential relationship was found for each examination, indicating that the tube current was successfully modulated according to the section size at each location within a given examination. However, a discrepancy between WEDslice and tube current was found in the caudal region: the tube current was lower than expected from WEDslice, which is explained by the application of ODM. In this study, ODM was applied over the orbit in all examinations to reduce the radiation dose to the eye lens and consequently the risk of radiation-induced cataracts. Organ tube current modulation, a similar function provided by Siemens (Erlangen, Germany), decreases radiation exposure from the anterior direction and increases that from the other direction [25]. In contrast, ODM provided by GE Healthcare decreases radiation exposure from the anterior direction and does not change that from the other direction [14,15], resulting in decreased radiation exposure per rotation.

When data from all slices of the 20 examinations were used for slice-based analysis, an exponential curve was well-fitted to the relationship between tube current and WEDslice. However, the tube current tended to be higher for slices with small WEDslice values than was predicted from the fitting curve. Excluding the most cranial slice of each image set improved exponential curve fitting. In this study, CT imaging was performed with a beam width of 10 mm and a slice thickness of 5 mm in the non-helical mode. The same tube current was applied to two contiguous slices, as shown in Figure 2b. Towards the top of the head, the section size and consequently WEDslice decreased rapidly, which appears to impair the relationship between tube current and WEDslice. The use of a wider beam width allows shortening of the imaging period but would further disturb tube current modulation, reflecting X-ray attenuation at each location faithfully.

A previous study investigating the relationship between radiation dose and age or weight in pediatric brain CT suggested higher radiation doses in male vs. female children at a given age or weight [16]. In the present study, CTDIvol was significantly larger in male than female patients after matching for age or weight, in line with the previous study, but not after matching for WEDmean. WEDmean was significantly larger in male patients after matching for age or weight. The sex-dependent differences in CTDIvol appear to be attributable to the large head size in male children at a given age or weight. It is indicated that radiation dose modulation according to the head size is achieved irrespective of sex.

The primary role of AEC is to aid in keeping image quality constant among patients and among slices. We evaluated the effect of AEC on image noise, using phantom experiments. The section size of the head phantom, represented by the WED, was smaller at the cranial location than at the middle location. When AEC was not used and tube current was unchanged along the scan range, image noise, represented by the SD, was definitely lower for the cranial slices than the middle slices. Using AEC, the tube current decreased for the cranial slices, leading to increased image noise. As a result, the difference in the image noise between slice locations was reduced. Although the phantom experiments in this study did not simulate imaging of different patients, the results support the effectiveness of AEC-based dose modulation in improving the consistency of image quality along the scan range.

Without AEC, the operator may determine the tube current by referring to the patient’s age or weight, and the determination may be based on the facility’s protocol or personal experience. A head size atypical for the age or weight may cause an inappropriate tube current setting. The possibility of an operator’s error is also a concern. AEC allows operator-independent tube current modulation that reflects the head size directly. Moreover, a fixed tube current value is applied without AEC to obtain an entire image set. The operator may set a relatively high tube current to avoid insufficient radiation exposure at strongly attenuating locations, resulting in excessive radiation at weakly attenuating locations. Although many facilities do not use AEC for pediatric brain CT [12,13], its use is recommended for radiation dose optimization.

There are different AEC systems provided by different manufacturers [23,26,27,28]. They show different behaviors, and the parameters to be set by the user are different. The characteristics of the AEC system should be recognized in each facility, and the appropriateness of the radiation dose and image quality should be confirmed. The main parameter of the AEC software used in this study is the noise index. This index represents the noise level of CT images reconstructed by filtered backprojection using a standard kernel. A higher noise index decreases radiation exposure and increases image noise. In this study, the noise index for pediatric brain CT was set at four, irrespective of age, a value higher than that for adult brain CT in our facility. It may be changed not only between children and adults but also among different age groups in children, considering different radiosensitivities depending on age. The slice thickness affects the noise level even when the same patient is imaged with the same radiation exposure, i.e., a higher noise level for a thinner slice. Image sets of different thicknesses may be reconstructed from given data simultaneously, and the AEC software determines tube current based on the primary image set. The slice thickness of the primary image set was 5 mm in all examinations in this study, and thinner slices were added when necessary. The slice thickness may be decreased in young children, resulting in increased radiation exposure at a given noise index, and alteration of the noise index may be considered.

There are other factors to be considered when using AEC. AEC determines tube current mainly based on the localizer image. The direction of localizer imaging has been shown to affect radiation dose in body regions [20,21,22,23]. Selection of the localizer direction, either the frontal or lateral direction, may also influence tube current modulation in brain CT. Reduction in radiation dose derived from the localizer imaging is desirable; however, excessive reduction may decrease contrast in the obtained image and disturb the estimation of X-ray attenuation [29]. We acquired two localizer images, posteroanterior and lateral images, using the minimum tube current applicable on the scanner. The posteroanterior image is used only for confirmation of appropriate positioning but not for tube current modulation, and may be omitted. Posteroanterior imaging is preferable to anteroposterior imaging to reduce the radiation dose to the eye lens. Centering of the patient’s head is important especially for tube current modulation using an anteroposterior or posteroanterior image [20,30,31]. When an anteroposterior localizer image is acquired with the head above the center, the head is magnified on the obtained image, resulting in an overestimation of X-ray attenuation and consequently causing excessive radiation exposure. Automatic positioning using artificial intelligence has been reported to decrease the effect of off-centering while shortening the positioning time [32].

In this study, CT scanners of one specification provided by one manufacturer were used. Investigation using AEC systems from other manufacturers remains to be performed. Moreover, although the image quality is accepted for diagnosis by radiologists in our clinical practice, we did not evaluate image quality systematically in this study, and assessed the appropriateness of dose modulation based on the relationship between the head size and radiation dose on the image-set basis and slice-by-slice basis. In the optimization process of radiological imaging, the balance between radiation dose and image quality is considered, and radiation dose is reduced while preserving clinical benefits offered by the imaging. To prove the appropriateness of AEC-based dose modulation, it is desired to assess whether AEC improves consistency in image quality among patients and among slices.

## 5. Conclusions

In this study, we investigated the relationship between the head size and radiation dose in pediatric brain CT performed using AEC. The phantom experiments showed that AEC decreased differences in image noise between slices of different section sizes. Image-set-based analysis of patient data demonstrated an exponential relationship between CTDIvol and WEDmean, and slice-based analysis showed exponential relationships between tube current and WEDslice, indicating successful radiation dose modulation according to the head size for each patient and for each slice location within a patient. Age-matched and weight-matched comparisons demonstrated higher CTDIvol in male than female patients, and the sex-dependent differences in radiation dose were attributable to those in WED. This study indicated the validity of AEC-based dose modulation in clinical situations. For the optimization of radiation doses, the application of AEC is recommended in pediatric brain CT.

## Figures and Tables

**Table 1:** Results of phantom experiments.

Imaging Condition	Cranial Location		Middle Location		Cranial-to-Middle Ratio
	Current (mA)	Noise	Current (mA)	Noise	
NI 2.5	303	2.38 ± 0.14	479	2.55 ± 0.15	0.93
NI 3	210	2.72 ± 0.16	332	2.89 ± 0.18	0.94
NI 3.5	154	3.06 ± 0.18	244	3.33 ± 0.15	0.92
NI 4	118	3.43 ± 0.22	186	3.70 ± 0.19	0.93
NI 4.5	93	3.84 ± 0.31	147	4.11 ± 0.24	0.93
NI 5	78	4.27 ± 0.29	119	4.55 ± 0.31	0.94
Fixed	180	2.86 ± 0.15	180	3.81 ± 0.21	0.75

NI 2.5 indicates imaging using AEC with a noise index of 2.5. Fixed indicates imaging using a fixed tube current. Image noise are presented as means ± standard deviations.

**Table 2:** Sex-dependent differences in CTDIvol and WEDmean.

Matched Parameter	CTDIvol (mGy)		*p*	WEDmean (cm)		*p*	*n*
	M	F		M	F		
Age	22.4	21.2	0.003	14.4	14.0	0.001	431
	(11.3–38.5)	(10.7–36.1)		(7.3–18.4)	(6.8–18.1)		
Weight	21.9	21.1	0.040	14.2	13.9	0.011	422
	(11.3–35.0)	(10.7–36.1)		(7.3–17.6)	(6.8–18.1)		
WEDmean	21.3	21.3	0.692	14.0	14.0	0.957	419
	(11.3–38.5)	(11.3–36.1)		(7.3–18.4)	(7.6–18.1)		

The medians (ranges) are presented. The *n* indicates the number of matched pairs.

## Data Availability

The data are available upon reasonable request from the corresponding author.
